# Development and validation of delirium prediction model for critically ill adults parameterized to ICU admission acuity

**DOI:** 10.1371/journal.pone.0237639

**Published:** 2020-08-19

**Authors:** Stephana J. Cherak, Andrea Soo, Kyla N. Brown, E. Wesley Ely, Henry T. Stelfox, Kirsten M. Fiest

**Affiliations:** 1 Department of Community Health Sciences, Cumming School of Medicine, University of Calgary, Calgary, AB, Canada; 2 Department of Critical Care Medicine, Cumming School of Medicine, University of Calgary, Calgary, AB, Canada; 3 O’Brien Institute for Public Health, University of Calgary, Calgary, AB, Canada; 4 Hotchkiss Brain Institute, University of Calgary, Calgary, AB, Canada; 5 Alberta Health Services, Calgary, AB, Canada; 6 PolicyWise for Children & Families, Calgary, AB, Canada; 7 Tennessee Valley Veteran’s Affairs Geriatric Research Education Clinical Center, Nashville, TN, United States of America; 8 Critical Illness, Brain Dysfunction, and Survivorship Center, Vanderbilt University Medical Center, Nashville, TN, United States of America; 9 Department of Psychiatry, Cumming School of Medicine, University of Calgary, Calgary AB, Canada; University of Colorado Denver, UNITED STATES

## Abstract

**Background:**

Risk prediction models allow clinicians to forecast which individuals are at a higher risk for developing a particular outcome. We developed and internally validated a delirium prediction model for incident delirium parameterized to patient ICU admission acuity.

**Methods:**

This retrospective, observational, fourteen medical-surgical ICU cohort study evaluated consecutive delirium-free adults surviving hospital stay with ICU length of stay (LOS) greater than or equal to 24 hours with both an admission APACHE II score and an admission type (e.g., elective post-surgery, emergency post-surgery, non-surgical) in whom delirium was assessed using the Intensive Care Delirium Screening Checklist (ICDSC). Risk factors included in the model were readily available in electric medical records. Least absolute shrinkage and selection operator logistic (LASSO) regression was used for model development. Discrimination was determined using area under the receiver operating characteristic curve (AUC). Internal validation was performed by cross-validation. Predictive performance was determined using measures of accuracy and clinical utility was assessed by decision-curve analysis.

**Results:**

A total of 8,878 patients were included. Delirium incidence was 49.9% (n = 4,431). The delirium prediction model was parameterized to seven patient cohorts, admission type (3 cohorts) or mean quartile APACHE II score (4 cohorts). All parameterized cohort models were well calibrated. The AUC ranged from 0.67 to 0.78 (95% confidence intervals [CI] ranged from 0.63 to 0.79). Model accuracy varied across admission types; sensitivity ranged from 53.2% to 63.9% while specificity ranged from 69.0% to 74.6%. Across mean quartile APACHE II scores, sensitivity ranged from 58.2% to 59.7% while specificity ranged from 70.1% to 73.6%. The clinical utility of the parameterized cohort prediction model to predict and prevent incident delirium was greater than preventing incident delirium by treating all or none of the patients.

**Conclusions:**

Our results support external validation of a prediction model parameterized to patient ICU admission acuity to predict a patients’ risk for ICU delirium. Classification of patients’ risk for ICU delirium by admission acuity may allow for efficient initiation of prevention measures based on individual risk profiles.

## Introduction

Delirium is a serious and distressing neuropsychiatric syndrome [1] frequently experienced by patients in the intensive care unit (ICU) [2]. Delirium is a multifactorial syndrome with acute onset that occurs in the context of illness and fluctuates throughout the day, due to complex interaction between predisposing and precipitating risk factors [3]. Characteristics of delirium include altered consciousness, inability to focus or shift attention [4]. Despite affecting up to 80% of critically ill patients [5], delirium is often underdiagnosed and undertreated [2]. Screening vulnerable patients for delirium is important to allow for early detection and prevention of ICU delirium [3].

Risk prediction models allow clinicians to forecast which individuals are at a higher risk for developing a particular outcome [6], and to implement interventions specific to the patients’ individual risk profile. An accurate delirium prediction model is therefore regarded as a powerful tool for an ICU clinician to facilitate early implementation of prevention measures [7]. Characterizing the risk profile associated with ICU delirium incidence for patients at their particular level of acuity at ICU admission might inform efforts to improve efficiency, value, and quality of ICU patient care. Compared to high acuity patients with more complex or serious conditions, low acuity patients in general require less monitoring, time for treatment and observation, and therefore shorter ICU stays.

Current guidelines recommend that ICU patients be routinely screened for delirium using either the Confusion Assessment Method-ICU (CAM-ICU) or the Intensive Care Delirium Screening Checklist (ICDSC) [8, 9]. Several delirium prediction models have been developed in response to recommendations from the Society of Critical Care Medicine Pain, Agitation/Sedation, Delirium, Immobility and Sleep clinical practice guidelines for routine use of delirium prediction models in daily ICU practice [10, 11]. Three prediction models have been validated to predict the risk for ICU delirium among critically ill delirium-free adults. The PRE-DELERIC [12] and the E-PRE-DELERIC [13] have been externally validated for use with both the ICDSC and the CAM-ICU, and the Lanzhou model [14] has been internally validated for use with the CAM-ICU. Another model, the dynamic Acute Brain Dysfunction-prediction model (ABD-pm) [15], has been internally validated using the CAM-ICU to predict next day status (i.e., normal, delirium, coma, ICU discharge or ICU death) among critically ill adults including those with delirium at time of ICU admission.

Although the PRE-DELERIC and the E-PRE-DELERIC have been validated for use with the ICDSC, no delirium prediction model exists that has been developed *using* the ICDSC. The ICDSC gathers information on the patient over 8 to 24 hours [16], which may lend the ICDSC to assessing the fluctuating course of delirium [17]. Further, because the CAM-ICU was specifically developed for use in non-verbal (i.e., mechanically ventilated) patients [18], the CAM-ICU may not accurately predict an episode of delirium in verbal patients with a low severity of illness if the specifically defined delirium features are not found at the time of assessment [19, 20]. Given that presentation of delirium in low severity patients is often more uncertain than in high severity patients, patients with a low severity of illness represent an important target to prevent incident ICU delirium [21]. Multiple studies have shown that the relationship between risk factors for ICU patient morbidity and mortality outcomes differ based on acuity of the ICU patient at the time of admission [22–25]. We propose that characterizing the relationship between risk factors for ICU delirium assessed by the ICDSC and based on admission acuity would further inform efforts to improve care aimed to prevent incident ICU delirium. Therefore, the aim of this study was to develop and internally validate a delirium prediction model for incident delirium using the ICDSC for delirium assessment parameterized to specific cohorts of patient ICU admission acuity.

## Materials and methods

### Ethical considerations

The study was approved by the University of Calgary Conjoint Health Research Ethics Board (REB17-0389) and a waiver of consent to access the data was granted. Since this was a retrospective cohort study using de-identified administrative data, obtaining consent from all participants was not feasible.

### Design and setting

This was a population-based retrospective observational cohort study investigating the ability of a delirium prediction model parameterized to seven patient cohorts to identify patients at risk of developing delirium during an ICU stay (i.e., parameterized cohort model). For clarity, comparison and clinical interpretation, we developed a general model inclusive of all patients (i.e. general inclusive model). Fourteen ICUs in a single-payer healthcare system across a single province in Canada were included (S1 Table). Included ICUs were mainly part of tertiary care (55.4%), teaching hospitals (83.1%), and primarily admitted as medical patients (62.6%), followed by surgical (24.7%) and neurological (5.6%) patients. All included ICUs use a single integrated electronic bedside clinical information system. The data sources and their linkage are described in supplemental methods in S1 File.

### Patients

This study consisted of consecutive critically ill adult patients (≥18 years) admitted to ICU between January 1, 2014 and June 30, 2016 who survived hospital stay. Patients were excluded if they had an ICU length of stay (LOS) <24 hours, died in the ICU or in the hospital immediately following ICU stay, did not have at least 1 assessment of delirium during their ICU stay, did not link to hospital admission data, were not a resident of the province of Alberta, were in sustained coma or delirious at ICU admission (determined respectively by the Glasgow Coma Scale [GCS] or a composite assessment of length of ICU stay, total number of ICDSC assessments, and number of ICDSC assessments ≥4), or if an Acute Physiology and Chronic Health Evaluation (APACHE) II score was not reported. Only the patient’s first admission to ICU during the study period was considered.

### Risk factors

Risk factors were considered for inclusion if they fulfilled five criteria: (1) previously identified in the literature, (2) available in clinical practice within 24 hours of patient admission to ICU, (3) were temporally observed before the occurrence of delirium, (4) able to administer without extensive training or interpretation, (5) did not exclude a more informative variable. For the parameterized cohort model, ten risk factors were identified according to these criteria and considered in the model development. The risk factors were: age, sex, APACHE II score at admission, GCS score at admission, SOFA (Sequential Organ Failure Assessment) score at admission, Charlson Comorbidity Index at admission, vasoactive medication receipt within 24 hours of ICU admission, pre-existing neuropsychiatric disorder (i.e., depression, anxiety, post-traumatic stress disorder or neurocognitive disorder, as these were groups identified in the literature to be commonly described after an ICU admission), continuous renal replacement therapy receipt within 24 hours of ICU admission, and invasive mechanical ventilation receipt within 24 hours of ICU admission. All scores (e.g., APACHE II, GCS, SOFA, Charlson Comorbidity Index) were included as either continuous or ordinal variables. Though the GCS score at admission was included as a standalone risk factor in all models, this score is also included in calculation of both APACHE II and SOFA scores. Dichotomous variables were age (≥65 years), sex (female), any use of vasoactive medication (i.e., dopamine, dobutamine, epinephrine, isoproterenol, milrinone, norepinephrine, phenylephrine or vasopressin), any pre-existing neuropsychiatric disorder (i.e., depression, anxiety, post-traumatic stress disorder or neurocognitive disorder), any requirement of continuous renal replacement therapy, and any requirement of invasive mechanical ventilation. No imputation techniques were used to handle missing data. The general inclusive model consisted of the ten risk factors, including an additional dichotomized risk factor for emergency admission to the ICU (i.e., all patients were included and were dichotomized either as emergent post-surgical admissions, or not [non-surgery and elective post-surgery admission]).

### Primary outcome

Prediction models predicted risk for delirium incidence during ICU stay as the primary outcome. ICU delirium was measured using the ICDSC [26], with reference to the Diagnostic and Statistical Manual of Mental Disorders [27], and can be administered quickly by a bedside clinician. In all ICUs in the province of Alberta, the ICDSC is a routine documentation as part of standard of care that is completed once every nursing shift (i.e., twice a day, regardless of day) by the bedside nurse. Additional detail on ICDSC delirium screening and determination of delirium subtypes are provided as supplemental material in S1 File.

### ICU admission acuity

Patient’s acuity at ICU admission was defined in two ways. First, we defined admission acuity by three categories of admission type: (1) elective post-surgery, (2) emergency post-surgery, and (3) non-surgical. Second, admission acuity was defined by creating quartiles of mean APACHE II score at admission for all patients. APACHE II is a validated ICU severity-of-illness adjustment system which is calculated based on the most severe (highest) value recorded during the first 24 hours in ICU for 12 physiologic variables and has shown to predict ICU and hospital mortality and LOS [28]. After confirming a near normal distribution, we categorized patient ICU APACHE II admission score into quartiles of low, medium, high, and highest acuity that promoted greater comparison of APACHE II scores [29]. The four quartiles were chosen to achieve four similar sized cohorts with quartile 1 representing APACHE II scores from 1 to 12; quartile 2, 13 to 17; quartile 3, 18 to 22; and quartile 4, 23 to 48.

### Statistical analysis

All analyses were conducted in Stata 15 Software (StataCorp. 2017. *Stata Statistical Software Release 15*. College Station, TX: StataCorp LLC). Baseline characteristics were summarized as medians with interquartile ranges (IQR) for continuous data and frequencies with proportions for categorical data. Model development followed rigorous methods outlined by Steyerberg [30] and Steyerberg and Vergouwe [31]. We used least absolute shrinkage and selection operator (LASSO) logistic regression [32] and adhered to iterative cycles of testing importance of predictors, assumptions of the model, and making subsequent adaptations. To validate the models using calibration and discrimination [33], we adhered to guidelines as outlined by the Transparent Reporting of a Multivariable Prediction Model for Individual Prognosis or Diagnosis (TRIPOD) Statement for statistical performance reporting on validation and clinical utility statistics [34, 35] and used ROC curve analysis (that reports an AUC) and measures of model accuracy [36]. Finally, predictive values and decision curve analysis were used to assess clinical utility of the models [37]. Additional detail on the data sources and statistical analyses are provided as supplementary material in S1 File.

## Results

**Fig 1:**
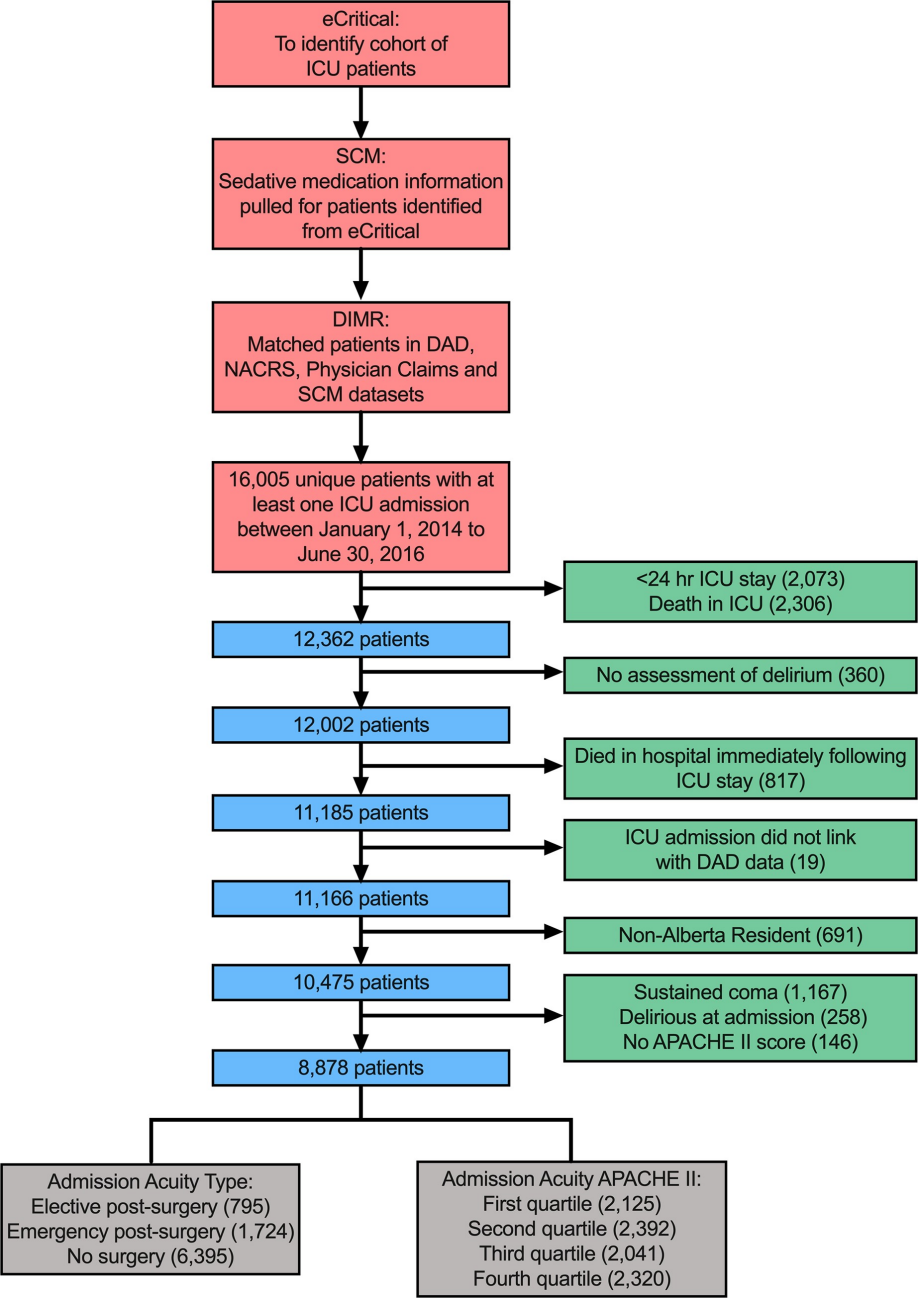
Data pull and participant flow. APACHE, acute physiology and chronic health evaluation; DAD, Discharge Abstracts Database; DIMR, de-identified medical record; NACRS, National Ambulatory Care Reporting System Metadata; SCM, Sunrise Clinical Manager.

During the study period 16,005 patients were admitted to one of the 14 ICUs. The final analysis cohort included 8,878 patients admitted to 14 ICUs in Alberta, Canada. The data pull and participant flow are illustrated in Fig 1. Demographics of the study cohort and characteristics of the participating ICUs are described as supplemental material in S1 File and shown in Table 1 and S1 Table, respectively. Half of the study cohort (49.9%) developed incident delirium during the study period (n = 4,431). S2 Table provides information on delirium presentations grouped by APACHE II quartiles and admission type. Characteristics of the delirium patient cohort are described as supplemental results in S1 File and in S3 Table, and S4 Table provides statistics on ICDSC assessments.

**Table 1:** Patient demographics.

		Admission Type			APACHE II Quartiles^5^			
Characteristic^1^	All patients	Elective post-surgery	Emergency post-surgery	Non-surgical	First Quartile	Second Quartile	Third Quartile	Fourth Quartile
Number of patients	8,878	795	1,724	6,359	2,125	2,392	2,042	2,320
Sex, female	3,765 (42.4)	325 (40.9)	748 (43.4)	2,692 (42.3)	812 (38.2)	1,027 (43.0)	906 (44.4)	1,020 (44.0)
Age	59 (46–69)	63 (54–72)	60 (45–71)	58 (45–69)	47 (33–59)	59 (47–69)	63 (52–73)	65 (55–73)
APACHE II score	17 (13–23)	15 (11–18)	16 (16–21)	18 (13–24)	10 (8–11)	15 (14–16)	20 (19–21)	26 (24–29)
GCS	15 (14–15)	15 (15–15)	15 (14–15)	15 (13–15)	15 (15–15)	15 (14–15)	15 (13–15)	14 (12–15)
SOFA score	6 (3–8)	4 (3–7)	6 (4–8)	6 (3–9)	3 (1–4)	5 (3–7)	6 (4–8)	9 (7–11)
Charlson Comorbidity Index	1 (0–3)	2 (1–4)	1 (0–2)	1 (0–3)	0 (0–1)	1 (0–2)	1 (0–3)	2 (1–3)
Pre-existing neuropsychiatric disorder^2^	5,302 (59.7)	427 (52.1)	862 (50.0)	4,025 (63.3)	1,226 (57.7)	1,425 (59.6)	1,224 (60.0)	1,427 (61.5)
Vasoactive medication use^3^	3,773 (42.5)	210 (26.1)	813 (47.2)	2,750 (43.2)	361 (17.0)	830 (34.7)	993 (48.7)	1,589 (68.5)
Continuous renal replacement therapy	407 (4.6)	7 (0.9)	49 (2.8)	351 (5.5)	4 (0.19)	20 (0.84)	54 (2.6)	329 (14.82)
Invasive mechanical ventilation	5,420 (61.1)	513 (64.5)	1,444 (83.8)	3,463 (54.5)	1,079 (50.8)	1,464 (61.2)	1,302 (63.8)	1,575 (67.9)
Delirium^4^	4,431 (49.9)	263 (33.1)	796 (46.2)	3,372 (53.0)	700 (32.9)	1,074 (44.9)	1,109 (54.3)	1,550 (66.8)
ICU length of stay (days)	4.0 (2.3–7.4)	2.4 (1.7–4.2)	3.6 (2.0–6.6)	4.4 (2.6–7.9)	2.7 (1.7–4.5)	3.5 (2.0–6.1)	4.6 (2.7–8.1)	5.9 (3.5–10.4)

APACHE, acute physiology and chronic health evaluation; GCS, Glasgow Coma Scale; SOFA, Sequential Organ Failure Assessment.

^1^Categorical data presented as frequency (%) and continuous data presented as median with interquartile range.

^2^I.e., depression, anxiety, post-traumatic stress disorder or neurocognitive disorder.

^3^I.e., dopamine, dobutamine, epinephrine, isoproterenol, milrinone, norepinephrine, phenylephrine or vasopressin.

^4^Patients who scored positive for delirium by ICDSC score ≥4 during ICU stay.

^5^Quartiles of mean APACHE II score for all patients admitted during a calendar year regardless of their risk profile.

The main ICU admission type was non-surgical (71.3%), followed by emergency post-surgery (19.4%) and elective post-surgery (9.0%) (Table 1). Patients with a non-surgical admission type had a median ICU length of stay of 4.4 days (2.6–7.9), which was longer than the median ICU length of stay for both post-surgery and elective post-surgery patients (3.6 [2.0–6.6] and 2.4 [1.7–4.2], respectively). Non-surgical patients had 6.8% and 19.9% greater delirium incidence compared to post-surgery and elective post-surgery patients, respectively. Similar findings were observed when admission acuity was determined by quartiles of mean APACHE II score (Table 1). Specifically, patients in the fourth quartile had 33.9% greater incidence of delirium and spent on average 3.2 more days in the ICU compared to patients in the first quartile of mean APACHE II score.

Table 2 presents the selected risk factors and their estimated coefficients. In general, risk factor coefficients that are large in terms of their absolute value have a greater influence on the prediction of delirium. S5 Table lists validity and accuracy statistics. Overall, specificity and the positive predictive value (PPV) were greater than sensitivity and the negative predictive value (NPV) within all patient cohorts.

**Table 2:** Model coefficients.

		Admission Type			APACHE II Quartile^6^			
Risk factor	All Patients	Elective post-surgery	Emergency post-surgery	Non-surgical	First Quartile	Second Quartile	Third Quartile	Fourth Quartile
Sex, female	-0.2437	NI	-0.3843	-0.2526	-0.4822	-0.1827	-0.1420	-0.1570
Age^1^	-0.0242	0.2214	0.0509	-0.1270	-0.2209	0.1413	-0.1196	-0.2054
Admission Type^2^	-0.2676	NI	NI	NI	NI	NI	NI	NI
APACHE II score^2^	0.0362	0.0202	0.0302	0.0300	0.0766	0.0130	-0.0879	0.0086
GCS^3^	-0.3202	-0.3857	-0.2851	-0.3698	-0.4790	-0.4411	-0.3458	-0.2714
SOFA score^3^	0.0524	0.0475	0.0610	0.0837	0.0820	0.0710	0.0879	0.0545
Charlson Comorbidity Index^3^	-0.0379	0.0262	-0.0634	-0.0472	-0.0239	-0.0719	-0.0357	-0.0410
Pre-existing neuropsychiatric disorder^4^	0.4880	0.3603	0.3714	0.5126	0.5765	0.4314	0.4015	0.4548
Vasoactive medication use^4^^,^^5^	0.1548	0.2414	0.4649	0.2572	0.1743	0.4651	0.2378	0.2817
Required continuous renal replacement therapy^4^^,^^5^	0.7895	1.0501	0.7393	0.7326	NI	1.0438	0.0859	0.7443
Required invasive mechanical ventilation^4^^,^^5^	0.9833	0.8849	0.6267	1.1313	1.1112	0.9455	0.7770	0.8879
Constant	2.7753	3.9561	2.8901	3.9692	5.0904	5.4772	4.6250	3.4013

APACHE, acute physiology and chronic health evaluation; GCS, Glasgow Coma Scale; NI, not included; SOFA, Sequential Organ Failure Assessment.

^1^Dichotomized to ≥65 years or <65 years.

^2^Dichotomized to emergency admission or non-emergency admission (i.e., non-surgical and elective post-surgery).

^3^Continuous variable.

^4^Dichotomized to yes or no.

^5^Receipt within 24 hours of ICU admission.

^6^Quartiles of mean APACHE II score for all patients admitted during a calendar year regardless of their risk profile.

**Fig 2:**
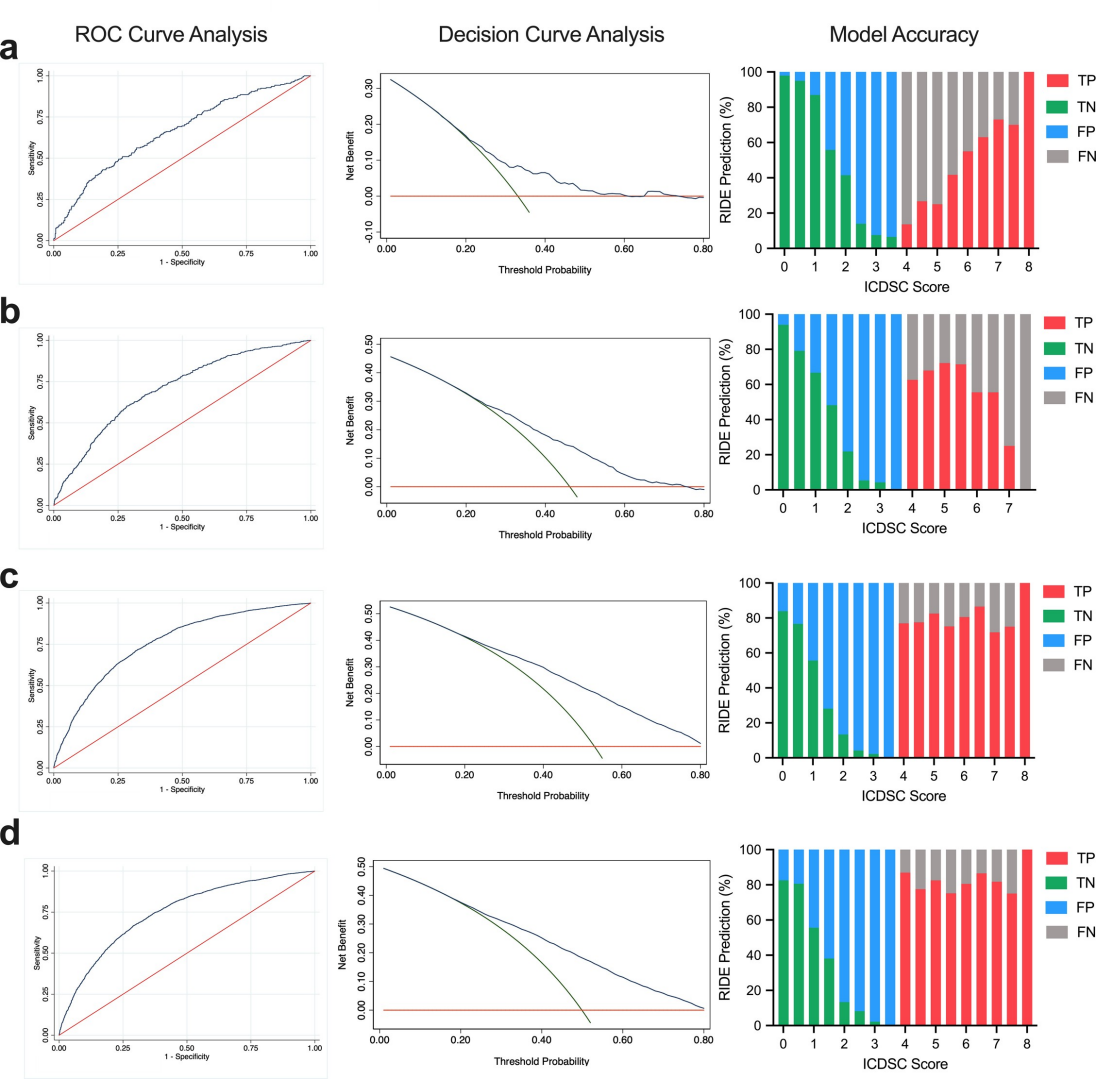
Model validity and accuracy for admission type. a, elective post-surgery, b emergency post-surgery, c non-surgical, and d all admission types. Left panel- The area under the ROC curve (AUC) is a measure of overall model performance and is interpreted as the average value of sensitivity for all possible values of specificity. Red line: relying purely on chance to discriminate between delirious and non-delirious patients. Blue line: ability of the model to discriminate. Middle panel- Decision curves for the prediction models. Red line: assume no patients are treated, net benefit is zero (i.e., no true positive and no false positive patients). Green line: assume all patients are treated. Blue line: patients are treated if predictions exceed a threshold. A risk prediction model is of clinical utility if the net benefit at a particular threshold probability is greater than treating all and none patients. Right panel- Ability of the prediction models to distinguish between a delirious and non-delirious patient (i.e., false negative, true negative, false positive, true positive) at increasing half-scores of ICDSC assessments.

**Fig 3:**
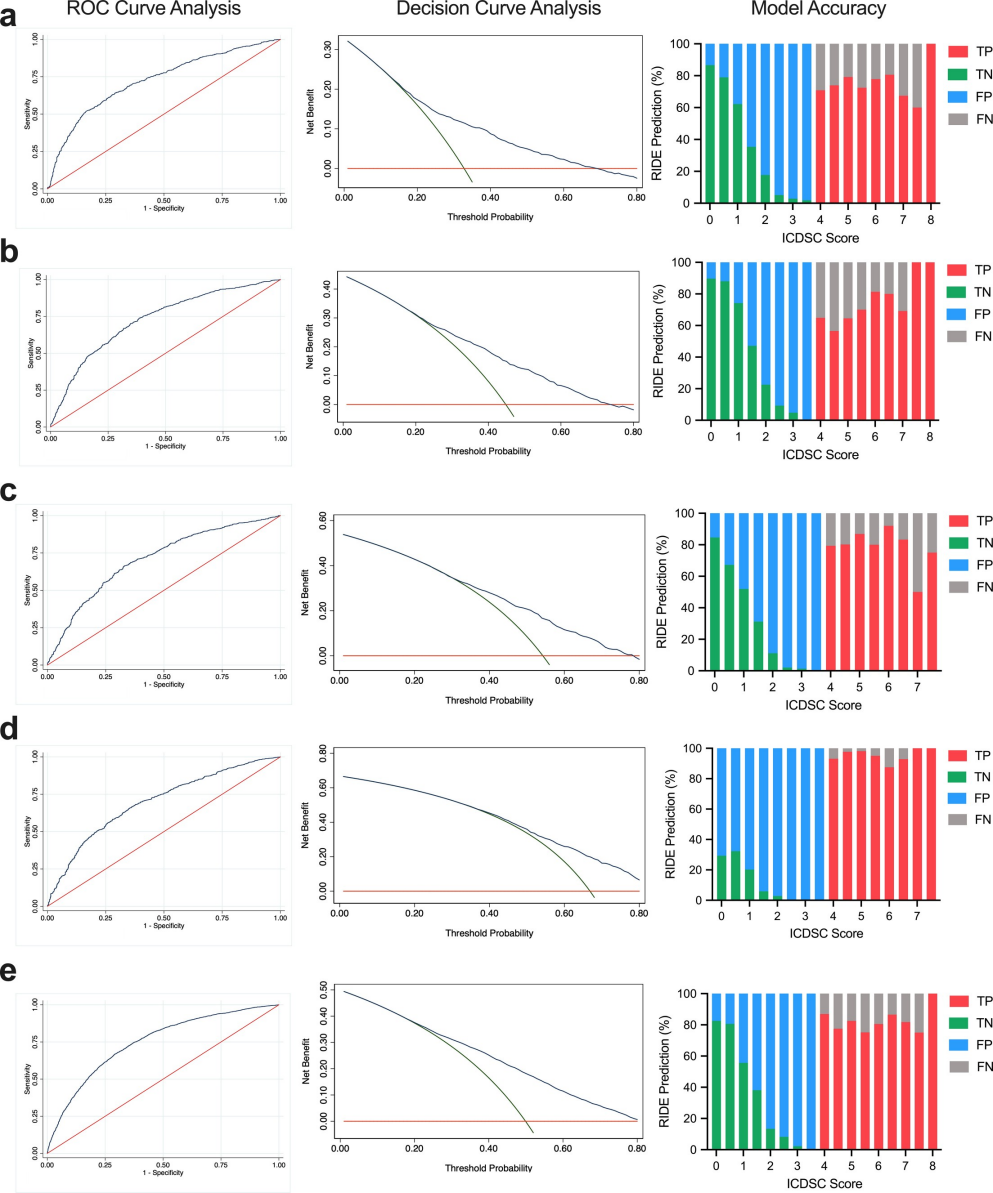
Model validity and accuracy for APACHE II quartile. a first quartile, b second quartile, c third quartile, d fourth quartile, and e all quartiles of mean APACHE II score. Left panel- The area under the ROC curve (AUC) is a measure of overall model performance and is interpreted as the average value of sensitivity for all possible values of specificity. Red line: relying purely on chance to discriminate between delirious and non-delirious patients. Blue line: ability of the model to discriminate. Middle panel- Decision curves for the prediction models. Red line: assume no patients are treated, net benefit is zero (i.e., no true positive and no false positive patients). Green line: assume all patients are treated. Blue line: patients are treated if predictions exceed a threshold. A risk prediction model is of clinical utility if the net benefit at a particular threshold probability is greater than treating all and none patients. Right panel- Ability of the prediction models to distinguish between a delirious and non-delirious patient (i.e., false negative, true negative, false positive, true positive) at increasing half-scores of ICDSC assessments.

Figs 2 and 3 depict graphical representations of the validity and accuracy statistics of the parameterized cohort model grouped by admission type, and by APACHE II quartile, respectively. The discriminative power (the area under the ROC [AUC]) for the entire cohort was 0.76 (95% confidence interval [CI] 0.75–0.77) and the sensitivity and specificity were 62.1% and 74.2%, respectively. In elective post-surgery patients admitted to the ICU the ROC was 0.67 (95% CI 0.63–0.70) with a sensitivity and specificity of 53.2% and 69.1%, respectively. This increased to 0.70 (95% CI 0.68–0.73), 61.1%, and 69.0%, for emergency post-surgery patients, and to 0.78 (95% CI 0.77–0.79), 63.9%, and 74.6%, for non-surgical patients. The model parameterized to patient cohorts by admission type had better accuracy (range: 0.66–0.69) and superior positive predictive values (range: 0.74–0.79) compared to other available prediction models for a single patient cohort.

The model parameterized to patient APACHE II quartiles showed comparable discriminative power (S5 Table). The validity and accuracy the parameterized cohort model grouped by APACHE II quartile is illustrated in Fig 3. The ROC for the first, second, third, and fourth quartiles was 0.76 (95% CI 0.74–0.78), 0.72 (95% CI 0.70–0.74), 0.70 (95% CI 0.67–0.72), and 0.70 (95% CI 0.67–0.72), respectively. Although ICU delirium incidence increased with increasing APACHE II quartile (i.e., 32.9% [95% CI 30.9–35.0] in the first quartile to 66.8% [95% CI 64.8–68.8] in the fourth quartile), the sensitivity and specificity remained similar (for the first, second, third, and fourth quartile: sensitivity- 59.3%, 59.7%, 58.2%, 59.3%, and specificity- 73.6%, 72.8%, 72.3%, 70.1%, respectively). The model parameterized to patient cohorts by APACHE II quartiles had better accuracy (range: 0.66–0.72) and superior positive predictive values (range: 0.76–0.78) compared to other available prediction models for a single patient cohort.

S6 Table lists model discrimination and calibration for the general inclusive model within patient cohorts grouped by admission type and APACHE II quartiles. Comparison of general inclusive model performance to parametrized cohort model performance determined as difference between statistic for general inclusive model to statistic for parameterized cohort model show near negligible difference in AUC (range of difference: -0.01 to 0.03).

To assess the temporal relationship between predicted delirium risk and a positive ICDSC delirium screen, we determined the median time to first ICDSC score ≥4 from patient ICU admission for true positive predictions from the general inclusive model within patient cohorts grouped by admission type and APACHE II quartiles. S7 Table shows the median time lead for true positives within all patients was 19.1 hours (IQR, 6.9–49.1). The earliest detection within cohorts of admission type was for non-surgical patients, 18.3 hours (IQR, 7.6–47.4), while the earliest detection within APACHE II quartiles was for patients within the first quartile, 16.9 hours (IQR, 6.1–45.7).

## Discussion

In this multi-center, population-based, retrospective cohort study, administrative data was used to develop a delirium prediction model parameterized to seven patient cohorts that predicted risk for ICU delirium based on ICU admission acuity. We demonstrated that a patient’s risk for incident delirium in the ICU can be accurately predicted using a prediction model parameterized to patient ICU admission acuity. When admission acuity was defined by admission type, the model parameterized to non-surgical patients displayed the best performance statistics compared to models parameterized to admission types. When admission acuity was defined by quartiles of mean APACHE II score, the model parameterized to patients in the first quartile performed better than models parameterized to patients in the second, third, and fourth quartiles. The parameterized cohort model did not display superior measures of diagnostic accuracy when compared to a general inclusive model for all patients which included an additional dichotomized risk factor for emergency admission to the ICU. Overall, the reported measures of diagnostic accuracy for the parameterized cohort model developed using the ICDSC were higher than the PRE-DELERIC [12], E-PRE-DELERIC [13], and the Lanzhou model [14], and were comparable to those reported from the ABD-pm to predict the next-day brain function state of delirium [15], but were not superior to a general inclusive model also developed using the ICDSC within the same retrospective patient population. External validation is needed and the PRE-DELERIC [12] and E-PRE-DELERIC [13] delirium prediction models should continue to be used in the meantime.

Although complementary to other delirium prediction models, the parameterized cohort model differs in important ways. Earlier prediction models considered admission acuity by including a risk factor corresponding to either emergency/urgent admission [12, 13], or emergency surgery [14]. As well the predictive performance of earlier prediction models indicated they function mainly to exclude a risk of delirium rather than to identify patients at high risk. For example, the ABD-pm developed by Marra and colleagues [15] demonstrated poor positive predictive value (0.55), meaning that the ability of the ABD-pm to predict incident delirium in the ICU is limited, and not to the level that might inform clinical decision making. The parameterized cohort model demonstrated better ability to accurately predict a true positive delirium outcome given a positive test result (PPV range: 0.74–0.79) within all parameterized patient cohorts compared to other available prediction models. However, it is difficult to attribute the fact that earlier prediction models demonstrated poor PPVs for delirium risk owing to exclusion of a risk factor more specific to admission acuity. Further, a better PPV would be expected for a highly prevalent disease, an important consideration given our cohort had a high delirium incidence. Notwithstanding, we demonstrate that a delirium prediction model parameterized to patient ICU admission acuity can accurately predict a patient’s risk for developing delirium in the ICU that may enable the clinician to guide earlier interventions to prevent incident delirium. The potential clinical benefit of the parameterized model is related to that delirium prevention strategies are more effective when implemented early after ICU admission [38].

We aimed to develop a model with the ability to guide targeted interventions to prevent delirium that are considered user-friendly by clinicians. An important aspect of clinical utility is clinician preference. Wassenaar and colleagues [13] reported a greater proportion of ICU clinicians found the E-PRE-DELERIC easier and quicker to use compared to the PRE-DELERIC. However, a majority of clinician respondents reported no difference between the two models and less than half reported regular use of a delirium prediction model in clinical practice. Indeed, a model that demonstrates good discrimination, calibration, and validation does not guarantee actual use of the model in clinical practice [39], nor does it guarantee the model will enhance medical decision making let alone improve health outcomes of targeted individuals [40, 41]. In fact the impact on health outcomes has only formally been studied for a very small number of available prediction models [30, 42–44], none of which are related to ICU delirium. We considered valuable lessons from several aspects of available prediction model impact studies. The parameterized cohort model was developed to adhere to clinician preference [45] by including risk factors previously identified in the literature and readily available within 24 hours of ICU admission, and able to be administered without extensive training or detailed interpretation. All risk factors were entered as either a continuous or an ordinal score, or as a dichotomized yes/no, to aid implementation of models in clinical practice [46]. External validation and additional model refinement of both the parameterized cohort model and general inclusive model will allow for more conclusive statistical recommendations regarding potential model selection and clinical operationalization.

Though the parameterized cohort model did not perform better than the general inclusive model, there are important applications to consider. Various ICU quality improvement initiatives have been widely recommended in the last decade [9]. Benchmarking is a reliable tool for ICU quality improvement [47]. Reproducible, risk-adjusted prediction models have strong potential to be robust targets for benchmarking projects [48] and are considered one of the next steps for ICU quality improvement [49]. Earlier studies have found that critical care patients with the same diagnosis and a comparable severity of illness have different delirium risk profiles, responses to treatment, and clinical outcomes [50, 51]. These differences make assessing meaningful variation in delirium incidence across ICUs challenging. The performance of the general inclusive model indicates ability to make predictions of a patient’s delirium risk across many demographics. The model parameterized to specific patient cohorts might allow for case-mix adjustment for delirium outcomes to ultimately enable objective comparisons of delirium outcomes across ICUs. Used for ICU benchmarking initiatives, the parametrized delirium prediction model will provide actionable information to improve patient outcomes.

In previous studies the assessment of delirium outcome was largely non-systematic, once daily, and avoided weekends [13, 38, 52]. In our study, the electronically captured ICDSC was performed twice daily including weekends by routine clinical staff in a large population-based sample, which reduced the potential for selection bias. Since all included ICUs are included in the same single-payer healthcare system, the ICDSC assessments are standardized. This systematic, frequent, and consistent assessment of delirium is a strength to our study. Furthermore, since the data required to calculate delirium risk were collected retrospectively, clinical staff performed ICDSC assessments without knowledge of patients’ risk for incident delirium which protected against assessment bias. Overall, all data included are available to calculate scores prospectively.

Our study has limitations. Though we reported a prediction model for the risk of delirium based on many demographic and clinical risk factors parametrized to several patient cohorts, we do not suggest our mathematical models can fully explain or predict the risk of delirium. It is likely that our use of the same number of parameters within stratified cohorts which resulted in increased model accuracy that was due to overfitting of the data. Further, there may be other important risk factors that were not considered in this study. For example, we did not examine hospital characteristics such as ICU staffing models [53], and therefore we cannot exclude the influence of staffing patterns on the predictive ability of our models. Instead we purport the model be used within the context of the individual patient to provide descriptive information for predicting delirium outcome, and to identify approaches aimed at preventing ICU delirium in a targeted manner.

We acknowledge the model included all ICU patients independent of delirium subtype. It is possible that models based on subtype of delirium may improve sensitivity and specificity. However, given that delirium subtypes are known to fluctuate within the course of delirium during ICU stay [54], models specific to delirium subtype are unlikely to be of great clinical use. As well, we did not restrict the number of patients included from any given ICU, which may have led to an overrepresentation of patients from one ICU in the overall dataset. Further, though we internally validated the models through cross-validation with bootstrapped 95% CI, we did not validate externally in a separate group of patients and therefore did not employ decision curve analysis comparisons next to gold standards or other delirium prediction models. This currently limits our ability to justifiably provide practical, clinical or statistical considerations regarding model selection or operationalization into clinical practice. We recommend external validation should be performed before any such distinctions are made.

Finally, though screening vulnerable patients for delirium is important for early detection and prevention [3], current evidence suggests multicomponent non-pharmacological strategies are most effective for delirium prevention [55]. The highly interdependent nature of component causes of delirium may prevent clinical separation for tailored interventions, complex interactions between causes produce synergistic effects which are lost when disaggregated. Though several pharmacological strategies for delirium management in ICU patients have been investigated [56], drug avoidance or reduction are the only supported pharmacological approaches; prevention strategies in general do not support pharmacological treatment in effectively preventing delirium onset [57, 58]. Development of targeted delirium strategies would make the use of delirium prediction models a valuable adjuvant to delirium prevention and management.

## Conclusions

We developed a risk prediction model for incident delirium using the ICDSC and parameterized to patient ICU admission acuity that showed superior statistical performance compared to other delirium prediction models. Classification of patients’ risk for ICU delirium by admission acuity may allow for efficient initiation of prevention measures based on individual risk profiles and improve understanding of risk factors that influence delirium outcomes for ICU patients across a range of admission acuity.

## Supporting information

S1 Table Hospital characteristics. (DOCX)

S2 Table Delirium incidence and subtypes for parameterized cohort model. (DOCX)

S3 Table Patient demographics by delirium incidence. (DOCX)

S4 Table ICDSC assessments. (DOCX)

S5 Table Model discrimination and calibration for parameterized cohort model developed to patient admission type and APACHE II quartiles. (DOCX)

S6 Table Model discrimination and calibration for general inclusive model within patient cohorts grouped by admission type and APACHE II quartiles. (DOCX)

S7 Table Median time to first ICDSC score ≥4 from ICU admission in hours for true positive predictions from general inclusive model. (DOCX)

S8 Table Model AUC (95% CI) for delirium incidence from secondary analyses for parameterized cohort model. (DOCX)

S1 File Supplemental methods and results. (DOCX)
