# Supplementary material for: Development and validation of delirium prediction model for critically ill adults parameterized to ICU admission acuity
Source: PLoS One. 2020 Aug 19;15(8):e0237639. doi: 10.1371/journal.pone.0237639 (PMC7437909; doi:10.1371/journal.pone.0237639)
Supplement: S1 Table — (DOCX) [file pone.0237639.s001.docx]

**S1 Table. Hospital characteristics^1^**

| Hospital | Number of patients | Sex, female | Age | Delirium^2^ | ICU length of stay (days) | Elective post-surgery | Emergency post-surgery | Non-surgical | APACHE II score^3^ |
| --- | --- | --- | --- | --- | --- | --- | --- | --- | --- |
| 1 | 202 (2.3) | 91 (45.1) | 59 (45-70) | 75 (36.6) | 3.7 (2.1-5.9) | 3 (1.5) | 16 (7.9) | 183 (90.6) | 16 (11-20) |
| 2 | 1,332 (15.0) | 518 (38.9) | 57 (42-68) | 782 (58.7) | 4.8 (2.7-9.2) | 134 (10.1) | 263 (19.7) | 935 (70.2) | 16 (12-21) |
| 3 | 529 (5.9) | 230 (43.5) | 62 (50-71) | 270 (52.7) | 3.4 (2.0-5.9) | 86 (16.3) | 127 (24.0) | 316 (59.7) | 19 (14-24) |
| 4 | 210 (2.4) | 93 (44.3) | 57 (41-67) | 70 (33.3) | 3.1 (1.9-5.0) | 12 (5.7) | 20 (9.5) | 178 (84.8) | 12 (9-17) |
| 5 | 435 (4.9) | 214 (49.2) | 63 (51-73) | 212 (48.7) | 4.5 (2.4-8.8) | 9 (2.1) | 67 (15.4) | 359 (82.5) | 19 (14-24) |
| 6 | 65 (0.8) | 21 (32.3) | 52 (46-57) | 14 (21.5) | 2.5 (1.7-3.9) | 1 (1.5) | 4 (6.2) | 60 (92.3) | 11 (9-15) |
| 7 | 959 (10.8) | 366 (38.2) | 60 (47-71) | 452 (47.1) | 4.9 (2.8-9.4) | 59 (6.2) | 180 (18.8) | 720 (75.1) | 17 (12-22) |
| 8 | 67 (0.8) | 33 (49.3) | 57 (42-67) | 26 (38.8) | 2.5 (1.7-4.9) | 6 (9.0) | 5 (7.5) | 56 (83.6) | 15 (10-20) |
| 9 | 1,712 (19.3) | 744 (43.5) | 58 (45-69) | 935 (54.6) | 3.4 (2.0-6.5) | 224 (13.1) | 427 (25.0) | 1,061 (62.0) | 17 (13-22) |
| 10 | 97 (1.1) | 46 (47.4) | 63 (55-72) | 60 (61.9) | 4.1 (2.0-6.9) | 11 (11.3) | 11 (11.3) | 75 (77.3) | 18 (11-24) |
| 11 | 621 (7.0) | 269 (43.3) | 61 (49-71) | 318 (51.2) | 3.8 (2.2-7.1) | 31 (5.0) | 127 (20.5) | 463 (7.6) | 19 (14-25) |
| 12 | 372 (4.2) | 181 (48.7) | 64 (53-75) | 161 (43.3) | 3.8 (2.0-6.7) | 19 (5.1) | 65 (17.5) | 288 (77.4) | 19 (14-24) |
| 13 | 522 (5.9) | 253 (48.5) | 58 (45-69) | 201 (38.5) | 4.9 (2.9-8.3) | 30 (3.8) | 63 (12.1) | 439 (84.1) | 16 (11-21) |
| 14 | 1,755 (19.8) | 706 (40.2) | 58 (45-68) | 847 (48.3) | 3.9 (2.3-7.1) | 180 (10.3) | 349 (19.9) | 1,226 (69.9) | 19 (14-24) |

APACHE, acute physiology and chronic health evaluation

^1^Categorical data presented as frequency (%) and continuous data presented as median with interquartile range

^2^Patients who scored positive for delirium by ICDSC score ≥4 during ICU stay

^3^Median APACHE II score for all patients admitted during a calendar year regardless of their risk profile
