# Supplementary material for: Development and validation of delirium prediction model for critically ill adults parameterized to ICU admission acuity
Source: PLoS One. 2020 Aug 19;15(8):e0237639. doi: 10.1371/journal.pone.0237639 (PMC7437909; doi:10.1371/journal.pone.0237639)
Supplement: S2 Table — (DOCX) [file pone.0237639.s002.docx]

**S2 Table. Delirium incidence and subtypes for parameterized cohort model**

|  | Parameterized Cohort Model | | | | | | |
| --- | --- | --- | --- | --- | --- | --- | --- |
|  | Admission Type | | | APACHE II Quartile^3^ | | | |
| Delirium | Elective post-surgery | Emergency post-surgery | Non-surgical | First Quartile | Second Quartile | Third Quartile | Fourth Quartile |
| Number of patients | 795 | 1,724 | 6,359 | 2,125 | 2,392 | 2,042 | 2,320 |
| Incidence^1^ | 33.1 (29.8-36.5) | 46.2 (43.8-48.6) | 53.0 (51.8-54.2) | 32.9 (30.9-35.0) | 44.9 (42.9-47.0) | 54.3 (52.1-56.6) | 66.8 (64.8-68.8) |
| Hyperactive^2^ | 44 (5.5) | 85 (4.9) | 369 (5.8) | 107 (5.0) | 158 (6.6) | 200 (4.9) | 133 (5.7) |
| Hypoactive^2^ | 118 (14.8) | 316 (18.3) | 1,166 (18.3) | 310 (14.6) | 358 (15.0) | 389 (19.1) | 543 (23.4) |
| Mixed^2^ | 91 (11.5) | 373 (21.6) | 1,768 (26.4) | 231 (10.9) | 510 (21.3) | 569 (27.9) | 832 (35.9) |
| No delirium^2^ | 532 (66.9) | 928 (53.8) | 2,987 (47.0) | 1,425 (67.1) | 1,319 (55.1) | 933 (45.7) | 770 (33.2) |
| Unable to classify^2^ | 10 (1.3) | 22 (1.3) | 159 (2.5) | 52 (2.5) | 47 (2.0) | 50 (2.5) | 42 (1.8) |

^1^Data presented as frequency % (SD)

^2^Data presented as frequency (%)

^3^Quartiles of mean APACHE II score for all patients admitted during a calendar year regardless of their risk profile
