# Supplementary material for: Development and validation of delirium prediction model for critically ill adults parameterized to ICU admission acuity
Source: PLoS One. 2020 Aug 19;15(8):e0237639. doi: 10.1371/journal.pone.0237639 (PMC7437909; doi:10.1371/journal.pone.0237639)
Supplement: S3 Table — (DOCX) [file pone.0237639.s003.docx]

**S3 Table. Patient demographics by delirium incidence**

| Characteristic^1^ | Delirium^4^ | No Delirium |
| --- | --- | --- |
| Number of patients | 4,431 | 4,447 |
| Sex, female | 1,788 (40.4) | 1,977 (44.5) |
| Age | 59 (46-69) | 59 (45-70) |
| APACHE II score | 19 (15-25) | 15 (11-20) |
| GCS | 14 (12-15) | 15 (15-15) |
| SOFA score | 7 (4-9) | 4 (3-7) |
| Charlson Comorbidity Score  Acute myocardial infarction  Congestive heart failure  Peripheral vascular disease  Cerebrovascular disease  Dementia  Chronic obstructive pulmonary disorder  Rheumatoid disease  Peptic ulcer disease  Mild liver disease  Diabetes  Diabetes with complications  Hemiplegia or paraplegia  Renal disease  Cancer  Moderate/severe liver disease  Metastatic cancer  AIDS | 1 (0-3)  300 (6.7)  503 (11.3)  224 (5.1)  236 (5.3)  103 (2.3)  897 (20.2)  75 (1.7)  175 (4.0)  250 (7.9)  628 (14.2)  818 (18.5)  111 (2.5)  253 (5.7)  442 (10.0)  177 (4.0)  124 (2.8)  17 (0.4) | 1 (0-3)  211 (4.7)  477 (10.7)  217 (4.9)  106 (2.4)  28 (0.6)  839 (18.9)  69 (1.6)  146 (3.3)  221 (5.0)  601 (13.5)  741 (16.7)  81 (1.8)  240 (5.4)  656 (14.8)  95 (2.1)  214 (4.8)  14 (0.3) |
| Vasoactive medication use^2^ | 2,310 (52.1) | 1,463 (32.9) |
| Pre-existing neuropsychiatric disorder^3^ | 2,930 (66.1) | 2,372 (53.4) |
| Requires continuous renal replacement therapy | 334 (7.5) | 73 (1.64) |
| Requires invasive mechanical ventilation | 3,332 (75.2) | 2,088 (47.0) |
| ICU length of stay (days) | 5.9 (3.4-10.8) | 2.9 (1.9-4.7) |

APACHE, acute physiology and chronic health evaluation; GCS, Glasgow Coma Scale; SOFA, Sequential Organ Failure Assessment

^1^Categorical data presented as frequency (%) and continuous data presented as median with interquartile range

^2^I.e., dopamine, dobutamine, epinephrine, isoproterenol, milrinone, norepinephrine, phenylephrine or vasopressin

^3^I.e., depression, anxiety, post-traumatic stress disorder or neurocognitive disorder

^4^Patients who scored positive for delirium by at least one ICDSC score ≥4 during ICU stay
