# Supplementary material for: Development and validation of delirium prediction model for critically ill adults parameterized to ICU admission acuity
Source: PLoS One. 2020 Aug 19;15(8):e0237639. doi: 10.1371/journal.pone.0237639 (PMC7437909; doi:10.1371/journal.pone.0237639)
Supplement: S4 Table — (DOCX) [file pone.0237639.s004.docx]

**S4 Table. ICDSC assessments**

| Statistic^1^ | All patients | Delirium | No Delirium |
| --- | --- | --- | --- |
| Total number of ICDSC assessments^2^ | 7 (4-14) | 11 (6-20) | 5 (3-9) |
| ICU length of stay (days) | 4.0 (2.2-7.3) | 5.8 (3.4-10.8) | 2.9 (1.9-4.7) |
| Median ICDSC score | 1 (0-3) | 3 (1-4) | 0.5 (0-1) |
| Number ICDSC assessments ≥4^3^ | 0 (0-3) | 3 (1-8) | NA |
| Calendar days with ICDSC assessment ≥4^4^ | 0 (0-2) | 2 (1-5) | NA |

ICDSC, Intensive Care Delirium Screening Checklist; NA, not applicable

^1^Continuous data presented as median with interquartile range

^2^ICDSC screening completed once per nursing shift; variation among delirium outcomes due to ICU length of stay

^3^I.e., total not consecutive number

^4^I.e., total not consecutive calendar days
