# Supplementary material for: Development and validation of delirium prediction model for critically ill adults parameterized to ICU admission acuity
Source: PLoS One. 2020 Aug 19;15(8):e0237639. doi: 10.1371/journal.pone.0237639 (PMC7437909; doi:10.1371/journal.pone.0237639)
Supplement: S5 Table — (DOCX) [file pone.0237639.s005.docx]

**S5 Table. Model discrimination and calibration for parameterized cohort model developed to patient admission type and APACHE II quartiles**

|  | Parameterized Cohort Model | | | | | | |
| --- | --- | --- | --- | --- | --- | --- | --- |
|  | Admission Type | | | APACHE II Quartile^5^ | | | |
| Statistic | Elective post-surgery | Emergency post-surgery | Non-surgical | First Quartile | Second Quartile | Third Quartile | Fourth Quartile |
| Delirium incidence^1^ | 33.1 (29.8-36.5) | 46.2 (43.8-48.6) | 53.0 (51.8-54.2) | 32.9 (30.9-35.0) | 44.9 (42.9-47.0) | 54.3 (52.1-56.6) | 66.8 (64.8-68.8) |
| Sensitivity^2^ | 53.2 | 61.1 | 63.9 | 59.3 | 59.7 | 58.2 | 59.3 |
| Specificity^2^ | 69.1 | 69.0 | 74.6 | 73.6 | 72.8 | 72.3 | 70.1 |
| PPV | 0.79 | 0.74 | 0.79 | 0.76 | 0.76 | 0.77 | 0.78 |
| NPV | 0.29 | 0.28 | 0.25 | 0.27 | 0.26 | 0.24 | 0.25 |
| Accuracy^3^ | 0.69 | 0.66 | 0.69 | 0.72 | 0.68 | 0.66 | 0.69 |
| AUC | 0.67 | 0.70 | 0.78 | 0.76 | 0.72 | 0.70 | 0.70 |
| Bootstrap bias corrected 95% CI, AUC | 0.63-0.70 | 0.68-0.73 | 0.77-0.79 | 0.74-0.78 | 0.70-0.74 | 0.67-0.72 | 0.67-0.72 |
| Hosmer-Lemeshow chi-squared p-value^4^ | 0.27 | 0.23 | 0.25 | 0.46 | 0.32 | 0.47 | 0.38 |

AUC, Area Under the ROC Curve; PPV, Positive Predictive Value; NPV, Negative Predictive Value

^1^Data presented as frequency % with standard deviation

^2^At population delirium incidence

^3^Accuracy is calculated as the total number of true positive predictions and true negative predictions divided by the total number of predictions

^4^The Hosmer-Lemeshow test assesses calibration of a logistic prediction model through sensitivity to the discrepancy between the model and real-world data

^5^Quartiles of mean APACHE II score for all patients admitted during a calendar year regardless of their risk profile
