# Supplementary material for: Development and validation of delirium prediction model for critically ill adults parameterized to ICU admission acuity
Source: PLoS One. 2020 Aug 19;15(8):e0237639. doi: 10.1371/journal.pone.0237639 (PMC7437909; doi:10.1371/journal.pone.0237639)
Supplement: S6 Table — (DOCX) [file pone.0237639.s006.docx]

**S6 Table. Model discrimination and calibration for general inclusive model within patient cohorts grouped by admission type and APACHE II quartiles**

|  |  | Performance of General Inclusive Model Within Patient Cohorts | | | | | | |
| --- | --- | --- | --- | --- | --- | --- | --- | --- |
|  |  | Admission Type | | | APACHE II Quartile^4^ | | | |
| Statistic | General Inclusive Model^5^ | Elective post-surgery | Emergency post-surgery | Non-surgical | First Quartile | Second Quartile | Third Quartile | Fourth Quartile |
| Delirium incidence^1^ | 49.9 (48.9-51.0) | 33.1 (29.8-36.5) | 46.2 (43.8-48.6) | 53.0 (51.8-54.2) | 32.9 (30.9-35.0) | 44.9 (42.9-47.0) | 54.3 (52.1-56.6) | 66.8 (64.8-68.8) |
| Sensitivity^2^ | 68.4 | 63.5 | 66.2 | 70.5 | 67.3 | 66.5 | 56.3 | 64.3 |
| Specificity^2^ | 71.4 | 56.7 | 63.18 | 71.7 | 68.5 | 70.5 | 77.1 | 86.3 |
| AUC | 0.77 | 0.66 | 0.70 | 0.78 | 0.76 | 0.75 | 0.73 | 0.72 |
| Bootstrap bias corrected 95% CI, AUC | 0.76-0.78 | 0.63-0.71 | 0.66-0.71 | 0.77-0.79 | 0.74-0.79 | 0.72-0.76 | 0.72-0.75 | 0.69-0.74 |
| Hosmer-Lemeshow chi-squared p-value^3^ | 0.18 | 0.52 | 0.15 | 0.98 | 0.23 | 0.45 | 0.53 | 0.13 |

| Comparison of General Inclusive Model Performance to Parameterized Cohort Model Performance | | | | | | | | |
| --- | --- | --- | --- | --- | --- | --- | --- | --- |
|  | General Inclusive Model^5^ | Admission Type | | | APACHE II Quartile | | | |
| Sensitivity^2*^ | NA | 10.3 | 5.1 | 6.6 | 8 | 6.8 | -1.9 | 5 |
| Specificity^2*^ | NA | -12.4 | -5.82 | -2.9 | -5.1 | -2.3 | 4.8 | 16.2 |
| AUC^*^ | NA | -0.01 | 0 | 0 | 0 | 0.03 | 0.03 | 0.02 |

AUC, Area Under the ROC Curve; NA, Not Applicable

^1^Data presented as frequency % with standard deviation

^2^At population delirium incidence

^3^The Hosmer-Lemeshow test assesses calibration of a logistic prediction model through sensitivity to the discrepancy between the model and real-world data

^4^Quartiles of mean APACHE II score for all patients admitted during a calendar year regardless of their risk profile

^5^General inclusive model developed and calibrated on entire patient population including risk factor for emergency or non-emergency ICU admission

^*^Determined as difference between the statistic calculated for the general inclusive model to the statistic calculated for the parameterized cohort model
