# Supplementary material for: Development and validation of delirium prediction model for critically ill adults parameterized to ICU admission acuity
Source: PLoS One. 2020 Aug 19;15(8):e0237639. doi: 10.1371/journal.pone.0237639 (PMC7437909; doi:10.1371/journal.pone.0237639)
Supplement: S7 Table — (DOCX) [file pone.0237639.s007.docx]

**S7 Table. Median time to first ICDSC score ≥4 from ICU admission in hours for true positive predictions from general inclusive model**

|  |  | General Inclusive Model Within Patient Cohorts | | | | | | |
| --- | --- | --- | --- | --- | --- | --- | --- | --- |
|  |  | Admission Type | | | APACHE II Quartile^3^ | | | |
| Statistic | General Inclusive Model^4^ | Elective post-surgery | Emergency post-surgery | Non-surgical | First Quartile | Second Quartile | Third Quartile | Fourth Quartile |
| Time^1^ | 19.1 | 19.6 | 19.5 | 18.3 | 16.9 | 18.4 | 19.2 | 19.6 |
| IQR^2^ | 6.87-49.1 | 6.30-54.7 | 7.62-47.4 | 6.72-50.1 | 6.10-45.7 | 6.70-49.0 | 6.60-51.9 | 7.58-51.5 |

^1^Median time in hours to first ICDSC score ≥4 following ICU admission

^2^Interquartile Range

^3^Quartiles of mean APACHE II score for all patients admitted during a calendar year regardless of their risk profile

^4^General inclusive model developed and calibrated on entire patient population including risk factor for emergency or non-emergency ICU admission
