# Supplementary material for: Development and validation of delirium prediction model for critically ill adults parameterized to ICU admission acuity
Source: PLoS One. 2020 Aug 19;15(8):e0237639. doi: 10.1371/journal.pone.0237639 (PMC7437909; doi:10.1371/journal.pone.0237639)
Supplement: S8 Table — (DOCX) [file pone.0237639.s008.docx]

**S8 Table. Model AUC (95% CI) for delirium incidence from secondary analyses for parameterized cohort model**

| ICU Acuity | Parameterized cohort model | Excluding COPD patients | Excluding diabetes patients with complications | Including total annual ICU volume as covariate | Including non-invasive mechanical ventilation as covariate |
| --- | --- | --- | --- | --- | --- |
| Total number of patients | 8,878 | 7,142 | 7,319 | 8,878 | 8,878 |
| Admission type  Elective post-surgery  Emergency post-surgery  Non-surgical | 0.67 (0.63-0.70)  0.70 (0.68-0.73)  0.78 (0.77-0.79) | 0.62 (0.58-0.67)  0.70 (0.68-0.73)  0.76 (0.75-0.77) | 0.62 (0.57-0.67)  0.70 (0.68-0.73)  0.75 (0.74-0.77) | 0.63 (0.58-0.66)  0.69 (0.67-0.72)  0.75 (0.74-0.76) | 0.66 (0.57-0.66)  0.71 (0.67-0.72)  0.76 (0.74-0.77) |
| APACHE II Quartile^1^  First Quartile  Second Quartile  Third Quartile  Fourth Quartile | 0.76 (0.74-0.78)  0.72 (0.70-0.74)  0.70 (0.67-0.72)  0.70 (0.67-0.72) | 0.72 (0.68-0.74)  0.72 (0.70-0.74)  0.72 (0.69-0.74)  0.70 (0.67-0.73) | 0.73 (0.69-0.74)  0.73 (0.71-0.75)  0.70 (0.68-0.73)  0.71 (0.68-0.74) | 0.73 (0.70-0.75)  0.73 (0.71-0.75)  0.70 (0.67-0.72)  0.71 (0.67-0.73) | 0.72 (0.69-0.74)  0.72 (0.70-0.74)  0.70 (0.68-0.73)  0.70 (0.67-0.72) |

COPD, chronic obstructive pulmonary disorder

^1^Quartiles of mean APACHE II score for all patients admitted during a calendar year regardless of their risk profile

^2^Determined as annual yearly mean of occupied beds at time of patient discharge from ICU
