# Supplementary material for: Development and validation of delirium prediction model for critically ill adults parameterized to ICU admission acuity
Source: PLoS One. 2020 Aug 19;15(8):e0237639. doi: 10.1371/journal.pone.0237639 (PMC7437909; doi:10.1371/journal.pone.0237639)
Supplement: S1 File — (DOCX) [file pone.0237639.s009.docx]

**Supplemental Methods**

**Data Sources**

Demographic, clinical, severity of illness, diagnostic, and outcomes data were prospectively captured electronically using administrative data from the eCritical Tracer Database, Discharge Abstract Database [DAD], National Ambulatory Care Reporting System [NACRS], and Physician Claims. The cohort was linked across to DAD, NACRS, and physician claims using unique lifetime identifiers for five years prior to ICU admission. eCritical is the primary source of critical care patient-level data in Alberta. This system allows for detailed clinical documentation and automated capture of device and laboratory data for all critically ill patients using a common system and documentation tools. eCritical is frequently used as a key data source for research initiatives and has been validated for research purposes [1]. The DAD captures data on all hospitalized patients, including dates of admission and discharge, and up to 25 diagnostic codes from the International Classification of Diseases, 10th revision, Canadian enhancement. NACRS captures patients who received emergency or ambulatory care and has up to 10 diagnostic codes from the Canadian Enhancement of International Statistical Classification of Diseases, 10^th^ Revision. Physician claims captures patients who received care from an outpatient clinical and has up to 3 fields for diagnostic codes from the Canadian Enhancement of International Statistical Classification of Diseases, 9^th^ Revision.

**Risk Factor Variables**

Neuropsychiatric disorders were identified through physician diagnosed ICD-10 codes that were captured in the DAD, NACRS, and Physicians Claims databases. Validated coding algorithms were used to identify depressive [2] and anxiety [3] disorders, and a coding list developed with the aid of a neuropsychiatrist was used to identify codes for trauma-and-stressor and neurocognitive disorders (Brown, K.N., Unpublished work).

**Delirium Screening and Subtypes**

The ICDSC checklist screens for delirium on eight different domains (i.e., level of consciousness, inattention, disorientation, hallucinations/delusions/psychosis, psychomotor agitation, inappropriate speech or mood, sleep wake/cycle disturbance, and symptom fluctuations) [4]. The ICDSC yields an ordinal score that can be further classify patients into domains of non-delirious (score 0-3) or delirious (score ≥4) [5]. In this study, a patient was determined to have developed delirium if they were assessed as ICDSC delirious (e.g., score ≥4) at any time during ICU stay. Delirium sub-types were classified based on criteria by Peterson et al. [6] using the Richmond Agitation-Sedation Scale (RASS) to determine level of consciousness and associated positive ICDSC score ≥4. The RASS is scored from -5 points (unarousable) to 0 points (calm) to +4 points (combative), where scores between -3 to 0 indicate hypoactive delirium, scores between 1 to 3 indicate hyperactive delirium, and scores that fluctuate between hypoactive and hyperactive indicate mixed delirium. All ICDSC scores ≥4 were linked to the closest RASS score within 4 hours of charting. If there was no RASS score documented within 4 hours of the ICDSC score, the sub-type was considered “unable to be classified”. If there was a RASS score within 4 hours of the ICDSC score but the RASS was -5, -4 or +4, the sub-type was considered “unable to be assessed”. If at least 1 assessment indicated hypoactive delirium and at least 1 assessment indicated hyperactive delirium, the patient’s sub-type was considered mixed.

**Model Development**

We aimed to develop a simple, robust model parameterized to specific patient cohorts that considered clinical knowledge and previous research, rather than relying on statistical selectional methods alone. For clarity, comparison and clinical interpretation, we also aimed to develop a general inclusive model for all included patients. To prevent overfitting to the available data (i.e., a model that may fit the data well but may not generalize to new patients outside the study cohort), we used LASSO logistic regression. This is a modelling technique that determines a shrinkage factor that is multiplied by the regression coefficients to provide a more reliable prediction for new patients. The delirium prediction models were developed on data from the first two-thirds of patients from all participating hospitals. In LASSO logistic regression, an ICDSC diagnosis of delirium was first entered as a dependent variable, Y, in the logistic regression model, and was coded as 0 for absent (ICDSC score <4) or 1 for present (ICDSC score ≥4). The probability of delirium incidence given the considered risk factors x_i_ was then calculated as follows:

$$P\left( Y=1 | x_{i} \right)=\frac{exp ({}_{0}+{}_{1}x_{i1}+\ldots+{}_{k}x_{\mathrm{ik}})}{1+exp ({}_{0}+{}_{1}x_{i1}+\ldots+{}_{k}x_{\mathrm{ik}})}$$

where x_i_=(x_i1_, x_i2_, …, x_ik_) are the risk factors of the ith observation. β_0_ is the intercept and β_j_ (j=1, …, k) is the coefficient corresponding to the jth risk factor. The LASSO logistic estimator β_0_, …, β_k_ was defined as the minimizer of the negative log likelihood subject to ${}_{j=1}^{k}| |$. Here, λ>0 is a tuning parameter (i.e., the shrinkage factor) that controls the sparsity of the estimator (i.e., the number of coefficients with a value of zero) and is selected in practice by cross-validation.

**Model Validation**

Calibration and discrimination were examined for validation of model predictive performance. Calibration assesses how closely the predicted probabilities reflect the actual risk of delirium. Cross-validation was used to select the LASSO logistic regression lambda (λ) and the final risk factors to be included in the models. The models were then calibrated on data from the last one-third of patients. To prevent the random split of data from having an influence on prediction performance, we repeated cross-validation and calibration 100 times using 100 different partitions of the dataset into random thirds. Discrimination was conducted after models demonstrated good calibration (i.e., Hosmer-Lemeshow chi-squared p-value >0.05). Discrimination assessed the ability of the model to distinguish between a delirious and non-delirious patient. Models with better discrimination have greater spread between predictions. To assess the discriminative power of the models, we used ROC curve analysis (that reports an AUC) and measures of model accuracy, such as true and false positive and negative values. Discrimination was deemed complete after all models displayed acceptable discriminative power at the threshold level of delirium incidence (i.e., AUCs ranged from 0.67 to 0.78) [7].

**Clinical Utility**

Metrics of diagnostic performance and model accuracy do not account for clinical utility of a prediction model (i.e., the ability to make better clinical decisions with a model than without) [8]. Therefore, decision curve analysis was performed to assess clinical utility. The clinical utility of decision curve analysis is that it accounts for preferences of health care providers across a range of threshold probabilities [9]. In decision curve analysis, a clinical judgment of the relative value of benefits (treating a true positive case) and harms (treating a false positive case) is used to calculate a decision analytic measure called the net benefit. The net benefit puts benefits and harms on the same scale, and a single net benefit is calculated for each possible threshold probability. The decision curve of a model is then developed by plotting the net benefits against the threshold probabilities and is compared to extreme cases of treating all or none patients. A risk prediction model is of good clinical utility if the net benefit at a particular threshold probability is greater than treating all and none patients.

**Secondary Analyses**

We performed several secondary analyses on all parameterized models. First, we excluded chronic obstructive pulmonary disease (COPD) patients and diabetes patients with complications, since these patients represented the majority of ICU LOS outliers (defined as >99th percentile). Second, we evaluated the role of total annual ICU patient volume determined as annual yearly mean of occupied beds at time of patient ICU discharge by including it as a risk factor in the models. Third, we included a risk factor to specify non-invasive mechanical ventilation (in addition to the risk factor that specifies invasive mechanical ventilation). Comparisons between secondary analyses was done using the AUC with a bootstrap corrected 95% CI.

**Supplemental Results**

**Patient Characteristics**

Of the 8,878 patients, the majority were male (57.6%), the median age was 59 (IQR 46-69) years, and 61.1% required invasive mechanical ventilation. At admission, the median APACHE II score was 17 (IQR 13-23), and the most common diagnosis was COPD (39.1%), followed by diabetes with complications (35.2%), and diabetes (27.7%). Median ICU LOS was 4.1 (IQR 2.3-7.7) days. Patients with incident delirium more frequently required vasoactive medication (52.1% vs. 32.9%), continuous renal replacement therapy (7.5% vs. 1.6%), invasive mechanical ventilation (75.2% vs. 47.0%) and had a pre-existing neuropsychiatric disorder (66.1% vs. 53.4%) compared to those who did not develop delirium in the ICU. Delirium patients also spent on average 3 days longer in the ICU than non-delirium patients. The median number of ICDSC assessments between delirium and non-delirium patients were 11 (IQR 6-20) and 5 (IQR 3-9), respectively, with median scores of 3 (IQR 1-4) and 0.5 (IQR 0-1), respectively. The median number of total calendar days with an ICDSC assessment ≥4 in patients who developed ICU delirium was 2 (IQR 1-5) (S4 Table).

**Risk Factor Variables**

The parameterized cohort models consisted of ten risk factors: age, sex, APACHE II score at admission, GCS score at admission, SOFA score at admission, Charlson Comorbidity Index at admission, vasoactive medication receipt within 24 hours of ICU admission, pre-existing neuropsychiatric disorder (i.e., depression, anxiety, post-traumatic stress disorder or neurocognitive disorder), continuous renal replacement therapy receipt within 24 hours of ICU admission, and invasive mechanical ventilation receipt within 24 hours of ICU admission. The general inclusive model consisted of the ten risk factors, including an additional risk factor for emergency admission to the ICU.

**Secondary Analysis**

Supplemental Table 6 presents results of our secondary analyses in the parameterized cohort models. In restricted analyses excluding COPD patients (N=1,736) or diabetes patients with complications (N=1,559) (who comprised the majority of LOS outliers in the study cohort), there was no change in model results and these patients were included in the final models. As well, neither the addition of a risk factor for total annual ICU patient volume nor the addition of a risk factor for non-invasive mechanical ventilation changed model results.

**References**

1. Brundin-Mather R, Soo A, Zuege DJ, Niven DJ, Fiest K, Doig CJ, et al. Secondary EMR data for quality improvement and research: A comparison of manual and electronic data collection from an integrated critical care electronic medical record system. J Crit Care. 2018;47:295-301. Epub 2018/08/14. doi: 10.1016/j.jcrc.2018.07.021. PubMed PMID: 30099330.

2. Fiest KM, Jette N, Quan H, St Germaine-Smith C, Metcalfe A, Patten SB, et al. Systematic review and assessment of validated case definitions for depression in administrative data. BMC Psychiatry. 2014;14:289. Epub 2014/10/18. doi: 10.1186/s12888-014-0289-5. PubMed PMID: 25322690; PubMed Central PMCID: PMCPMC4201696.

3. Marrie RA, Walker JR, Graff LA, Lix LM, Bolton JM, Nugent Z, et al. Performance of administrative case definitions for depression and anxiety in inflammatory bowel disease. J Psychosom Res. 2016;89:107-13. Epub 2016/09/25. doi: 10.1016/j.jpsychores.2016.08.014. PubMed PMID: 27663119.

4. Pun BT, Ely EW. The importance of diagnosing and managing ICU delirium. Chest. 2007;132(2):624-36. Epub 2007/08/19. doi: 10.1378/chest.06-1795. PubMed PMID: 17699134.

5. Bergeron N, Dubois MJ, Dumont M, Dial S, Skrobik Y. Intensive Care Delirium Screening Checklist: evaluation of a new screening tool. Intensive Care Med. 2001;27(5):859-64. Epub 2001/06/30. PubMed PMID: 11430542.

6. Peterson JF, Pun BT, Dittus RS, Thomason JW, Jackson JC, Shintani AK, et al. Delirium and its motoric subtypes: a study of 614 critically ill patients. J Am Geriatr Soc. 2006;54(3):479-84. Epub 2006/03/23. doi: 10.1111/j.1532-5415.2005.00621.x. PubMed PMID: 16551316.

7. Hosmer DW, Lemeshow S. A goodness-of-fit test for the multiple logistic regression model. Communications in Statistics. 1980;A10:1043-69.

8. FitzGerald LZ, Rorie A, Salem BE. Improving secondary prevention screening in clinical encounters using mhealth among prelicensure master's entry clinical nursing students. Worldviews Evid Based Nurs. 2015;12(2):79-87. Epub 2015/02/24. doi: 10.1111/wvn.12081. PubMed PMID: 25704142.

9. Vickers AJ, Van Calster B, Steyerberg E. Decision Curves, Calibration, and Subgroups. J Clin Oncol. 2017;35(4):472-3. Epub 2017/01/28. doi: 10.1200/JCO.2016.69.1576. PubMed PMID: 28129527.
